# Socioeconomic gaps in science achievement

**DOI:** 10.1186/s40594-018-0132-5

**Published:** 2018-10-10

**Authors:** Laura Betancur, Elizabeth Votruba-Drzal, Christian Schunn

**Affiliations:** Department of Psychology & Learning Research and Development Center, University of Pittsburgh, 210 South Bouquet Street, Pittsburgh, PA 15260 USA

**Keywords:** Science achievement, Development, Socioeconomic gaps, Parental income, Parental education

## Abstract

**Background:**

In contrast to the extensive research on socioeconomic gaps in reading and math achievement, little attention has been given to socioeconomic disparities in science skills, particularly during the early years of schooling. This emphasis on later years may be problematic because large socioeconomic disparities emerge in the early years, thus it is crucial to document the size of disparities in science achievement and begin unpacking the range of factors that contribute to these disparities. Additionally, it is crucial to know which components of socioeconomic status are more strongly linked to children’s science skills so that resources can be more effectively targeted to address disparities. Using nationally representative data from the Early Childhood Longitudinal Study, Kindergarten Class of 1998-99 (*N* = 9250), this study examines disparities in science achievement across elementary and middle school related to parental income and parental education separating their effects from each other and from a range of confounding factors. Additionally, it considers whether characteristics of children, families, and schools are pathways through which socioeconomic disparities emerge.

**Results:**

Results show moderate gaps in science achievement related to both household income and parental education*.* The primary pathways through which parental education and family income influenced science achievement was through mathematics and reading achievement. For parental education gaps, smaller indirect effects also operated through access to informal science learning opportunities both inside and outside of the home environment.

**Conclusion:**

First, this study highlights the importance of considering the contributions of multiple measures of socioeconomic status, instead of a composite. Second, it shows that socioeconomic disparities in science achievement emerge early and that programs and policies aimed at addressing these gaps may need to target children during the early elementary and preschool years. Third, our findings suggest that elementary instructional approaches that simultaneously address science instruction with reading and/or mathematics instruction will likely be especially important for improving overall science outcomes.

## Background

Improving the science achievement of students in the U.S. has been a central focus for policy makers and researchers for decades (Gonzalez and Kuenzi 2012; Kuenzi 2008; National Research Council 2012a). It has become even more important in recent years because employment in science-related professions is expected to increase more than in other occupations (Hanson and Slaughter 2016; National Science Board 2012; Wang 2013). Growing concern has been expressed about disparities in science achievement between children from socio-economically disadvantaged households and their more advantaged counterparts (Riegle-Crumb and King 2010). These gaps have important implications for access to professional and technical careers in science, technology, engineering, and mathematics (STEM) as well as in health professions (Cannady et al. 2017). Yet, surprisingly little attention has been paid to disentangling socioeconomic disparities in science achievement (Ma 2001; Muller et al. 2001), despite extensive literature addressing socioeconomic gaps in reading and math skills (e.g., Votruba-Drzal 2006; Votruba-Drzal et al. 2013).

Additionally, the little attention that has been given to socioeconomic gaps in science achievement has focused on high school, with few studies documenting the emergence of these gaps during elementary and middle school (Quinn and Cooc 2015; Morgan et al. 2016). This emphasis on high school may be problematic because large socioeconomic status (SES) disparities emerge in academic domains in the early years (Duncan et al. 2011, 1998) and it is crucial to document the early emergence of socioeconomic disparities in science achievement and explore the range of factors that contribute to these disparities. By the time youth reach high school, it may be more difficult to address these deeply entrenched achievement disparities (Duncan et al. 1998; Duncan et al. 2010). In addition, a number of reports have pointed to the critical importance of strong early science education (National Research Council 2012a, 2012b). Using nationally representative data from the Early Childhood Longitudinal Study, Kindergarten Class of 1998-99 (ECLS-K: 99), this study examines the development of socioeconomic disparities in science achievement across third, fifth, and eighth grades. Additionally, it considers whether characteristics of children, families, and schools are pathways through which socioeconomic disparities emerge.

### Gaps in science by SES

Family SES reflects a family’s position in the social and economic hierarchy and the resources, prestige, and privileges that derive from this position (Hauser and Warren 1997). SES is commonly measured using a single indicator, such as household income or parental educational attainment, or with a composite measure that combines information across several indictors to reflect the multiple resources that shape the experiences of children and families at different levels of the social and economic hierarchy (Kohn 1963).

The number of studies focused on SES gaps in science achievement is small, particularly when compared to the exhaustive literature addressing gender gaps. The few studies that have considered SES gaps using composite measures have uncovered moderate links between SES and science skills (Ma 2001; Maerten-Rivera et al. 2010; Von Secker 2004; Von Secker and Lissitz 1999; Zhang and Campbell 2015). In the USA, Von Secker (2004) found that in fourth, eighth, and twelfth grades, low-SES students scored 0.69, 0.97, and 0.65 standard deviations below their higher SES peers. In a follow-up study of US tenth graders, Von Secker and Lissitz (1999) found that each standard deviation increase in SES was related to 0.44 standard deviation higher science achievement. In a large study of eighth grade students’ science performance in China, Zhang and Campbell (2015) found large SES gaps in science achievement with SES measured at the school level (i.e., high SES vs. low SES schools) and moderate gaps with SES assessed at the child level.

There are two major limitations in current knowledge of socioeconomic disparities in science achievement. First, this literature has focused heavily on older children, with particular attention to the factors associated with entering and persisting in a science-related career (e.g., Wang 2013). For example, SES predicts high school science achievement in part through access to high-quality education (Byrnes and Miller 2007). Generally, family income and SES more predict persistence in college (Witkow et al. 2015), in part because they have less social capital and are more likely to have a part time job (Walpole 2003). Thus, research has concentrated more heavily on achievement after high school or before college, and less attention has been paid to early stages of development, although there are some exceptions. However, studies focused on gender and race/ethnic gaps suggest that science achievement gaps emerge early (Quinn and Cooc 2015). This is a major shortcoming in the literature because early science learning lays the foundation for subsequent success in high school and beyond. Thus, it is crucial to examine the early emergence and development of disparities in science achievement in elementary school.

Second, studies considering links between SES and science achievement have typically assessed SES with a composite measure (Morgan et al. 2016). Unfortunately, no studies to date have systematically examined the emergence of achievement gaps in science by components of SES (e.g., by parental income and parental education) from elementary school up to the end of middle school. There are several limitations to composite measures of family SES that combine multiple dimensions of SES. First, although these components are consistently correlated, these correlations are only moderate (e.g., Davis-Kean, 2005). Second, as Duncan and Magnuson (2003) highlight, components of SES have distinct relations with children’s development and these associations are not interchangeable. Combining multiple SES indices into a single composite treats these as though they are interchangeable and does not allow for the careful examination of each pieces’ association with children’s development. Third, it is crucial to know which components of SES are mostly strongly linked to children’s science skills so that resources can be more effectively targeted to address SES disparities. Without a more nuanced understanding of the role of each dimension of SES in shaping science achievement, it is difficult to target interventions to effectively narrow gaps.

### Factors that contribute to SES gaps in science achievement

Parental education is a key dimension of SES that shapes children’s academic skills development (see the Harding et al. 2015, for a theoretical discussion). Parental education may increase parents’ human, cultural, and social capital, which can influence their childrearing knowledge, practices, beliefs, and aspirations as well as their parenting skills related to children’s science learning (Davis-Kean 2005; Hoff 2003; Raviv et al. 2004). For example, more educated parents promote child achievement by holding high educational expectations for their children, by providing more stimulating materials and activities, engaging in complex conversation, and providing higher quality of instruction (Davis-Kean 2005; Hoff 2003, Raviv et al. 2004). Parental education also can influence the time that parents have to invest in children by shaping characteristics of their employment, such as the level of employment autonomy and flexibility. Furthermore, research has shown that parental education is related not only to the time that parents spend with their children but also the quality of that time, reflected in the complexity of activities and higher levels of cognitive stimulation provided during those times (Kalil et al. 2012).

By contrast, family income tends to influence child’s academic development through investments in children and by reducing the levels of family stress (Duncan et al. 2017). Important investments include those made in the home learning environment. Income affects the amount, type, and quality of material goods, services, and activities/experiences parents invest in children. For example, parents with higher income can access better quality education, books, learning materials, summer camps, and more extracurricular activities than low-income parents (Kaushal et al. 2011). Additionally, high family income lessens the economic stress related to paying bills and purchases goods and services. Chronic stress threatens the development of child’s self-regulation and attentional skills directly (Knudsen et al. 2006) and parenting in the context of high family stress tends to be harsh and more detached as well as less responsive and nurturing (Kessler and Cleary 1980; McLeod and Kessler 1990). Beyond the family, income also shapes neighborhood and school contexts that children experience (Evans 2004).

Though correlated, evidence shows income and education are independently related to children’s development (Chevalier et al. 2013; Erola et al. 2016). The effects of income typically decline after controlling for parental education/cognitive skills (e.g. Blau 1999). The effects of parental education persist after controlling for income but also are typically larger than the effects of income on child outcomes (Chevalier 2004; Chevalier et al. 2005, 2013; Reardon 2011). From a policy perspective, it is crucial to consider the associations between components of SES and children’s science skills so that interventions can be targeted more effectively to address science achievement disparities. Some have argued that parental education should be targeted to reduce socioeconomic disparities in achievement because it will be more effective in creating permanent changes to children’s opportunities for learning. Indeed, some studies find stronger associations between parental education when compared to concurrent family income (Cameron and Heckman 1998; Carneiro and Heckman 2003; Chevalier and Lanot, 2002).

Therefore, it is important to isolate the direct associations of family income and parental education with achievement gaps, but also to unpack the indirect effects that may give rise to disparities in science achievement. A variable that is frequently named as underlying SES effects on educational achievement is access to high-quality education (Aikens and Barbarin 2008). In elementary school, differences in the quality of science education might involve the degree to which science is included in school at all in the school curriculum or the extent to which the teachers have training in science (Nasir et al. 2011). Schools serving low SES populations frequently have shortages of teachers who are fully qualified in science (Muijs et al. 2004; Nasir et al. 2011). Instead, elementary and sometimes even middle schools may use homeroom teachers for teaching science (Nasir et al. 2011). In a large study in eighth grade in China, Zhang and Campbell (2015) found that, relative to high SES schools, low SES schools were less likely to have high-quality teachers, defined in terms of having an undergraduate degree in the science area being taught, number of years teaching the given science topic, and level of the highest degree.

The home learning environment may provide experiential opportunities for science learning that undergird socioeconomic differences in science skills as well (Orr 2003). Additionally, higher SES families may provide greater access to after-school science enrichment activities or science camps during the summer. In the USA, the difference in annual enrichment spending by parents in the top and bottom income quintiles nearly tripled from 1972 to 2006 (Duncan and Murnane 2011). Additionally, opportunities to engage in more informal science learning activities at home (e.g., reading books about dinosaurs or using science kits) may be important as well (Barron et al. 2009). Such experiences may provide greater supporting knowledge for later science learning or increase the perceived value of learning science (Orr 2003). Thus, there are a variety of mechanisms by which SES differences in home support for science learning could also cause SES disparities in overall science achievement.

Additionally, SES gaps in other domains of academic achievement may give rise to disparities in science achievement. Reading and math skills tend to be strongly related to science achievement (Morgan et al. 2016; Quinn and Cooc 2015). Extant literature documents socioeconomic disparities in reading and math skills (for review see Duncan et al. 2015), thus SES disparities in science may, in part, be caused by disparities in mathematics and reading (Morgan et al. 2016). There is some evidence to suggest that reading skills may have larger effects on science achievement than do mathematics skills (Gustin and Corazza 1994; Maerten-Rivera et al. 2010). Relatedly, studies examining the effectives of enhancing reading skills (Voss and Silfies 1996) and reading strategies (Cottrell and McNamara 2002; O'Reilly and McNamara 2007) show benefits for science comprehension.

Beyond these mechanisms, it is important to understand whether SES gaps are true differences related to SES or whether they simply reflect linkages between SES and other factors that are also associated with science achievement, such as race/ethnicity (Norman et al. 2001), family structure (Ma 2001), and English language learning status (Abedi 2002; Santau et al. 2011; Maerten-Rivera et al. 2010). Also, although not previously examined in science, rural areas tend to have lower SES families and lower general academic achievement (Albrecht and Albrecht 2004; Burton et al. 2013); thus, urbanicity may be another important correlated factor. Past studies of SES gaps in science achievement have not controlled for other characteristics of families that may have a confounding influence. Thus, it is difficult to know whether observed gaps are attributable to other correlated factors.

Using nationally representative data from the Early Childhood Longitudinal Study, Kindergarten Class of 1998-99 (ECLS-K: 99), this study aims to strengthen knowledge of socioeconomic disparities in science achievement by separating the links with income and parental education and by removing confounds with a range of correlated factors including ethnicity, urbanicity, home language, and family structure. A central focus of this investigation is the magnitude and stability of socioeconomic gaps in science skills across third, fifth, and eighth grades. Furthermore, it considers whether SES gaps in science achievement are explained by differences in early learning opportunities related to family and school contexts. Finally, this investigation examines whether links between SES and science achievement are explained by differences in children’s reading and math achievement.

We hypothesize that income and education will independently relate to children’s science skills during elementary school and will operate through learning opportunities in the home environment, the quality of science education in school contexts, and children’s reading and math skills. We expect that parental education will be more strongly associated with early learning opportunities for science, whereas income would be strongly associated with the quality of science education in school contexts. Coming to understand the extent to which SES gaps in science achievement are grounded in high-quality science instruction, differences in home support for science learning, or differences in mathematics and reading achievement has important implications for policy since each of those factors requires different forms of interventions.

## Method

### Sample

The study draws data from the ECLS-K, a longitudinal, nationally representative, and multimethod study that focuses on US children’s early school experiences from kindergarten through eighth grade. There are two central strengths of the ECLS-K data. First, it is nationally representative of US children attending kindergarten during the 1998–1999 school year and eighth grade in the 2007–2008. Second, the study includes repeated and consistent measures of parent and home information, child development, and classroom characteristics, which allows us to test consistent models of children’s science achievement across grades. Given that the ECLS-K dataset is publicly available and de-identified, the Institutional Review Board of the University of [masked for review] declared that the research used in this manuscript was exempt.

This study focuses on a subsample of about 9250 children followed by the ECLS-K from kindergarten to eighth grade, who were clustered in 2700 schools. On average, there are 10.1 (SD = 5.1) children per school and 4.2 (SD = 3.4) children per classroom. Intraclass correlations were estimated for third (0.35), fifth (0.34), and eighth grades (0.36). Among these children, 64% had complete data on the large number of variables included in the analyses. Of cases without complete data, 18% had missing data on one variable, and the greatest number of missing variables for any one participant was 9 (18.75% of the variables and 0.1% of participants). The percentage of missing data for each variable in the analyses ranged from 0.2 to 16%. The percentage of missing data varied depending on the source of information: from 0 to 4.5% in the invariant child characteristics, from 0.6 to 5.1% in the children academic assessment, from 1.5 to 14.6% in the household characteristics questionnaire, and from 2.7 to 16.1% in the school and science teacher questionnaires. Missing data were addressed using full information maximum likelihood, which is considered the best approach to handling missing data (Allison 2002). A sampling weight (C7CW0) was utilized to adjust for differential sampling and attrition, so the results are generalizable to the nationally representative kindergarten cohort.

### Measures

The dataset contains a large number of variables. To avoid obtaining spurious findings by chance or under-powering the analyses through corrections for by-chance findings across large numbers of variables, the analyses focused on the variables directly relevant to the research questions and grounded in prior research findings.

#### Science achievement

Science achievement was measured using direct assessments that consisted of 100 items in third, fifth, and eighth grades. The test includes two types of competencies. The first is conceptual understanding, which is factual knowledge about science and conceptual understanding for why things occur as they do. The second is scientific investigation skills which reflect children’s abilities to formulate, answer, and communicate scientific questions about the natural world. The test assesses knowledge in the fields of earth and space science, physical science, and life science (Najarian et al. 2009). The tests were based on curricular expectations for each grade. Thus, children were not administered the exact same questions at all ages. In addition, science assessments were delivered in a two-stage adaptive process. First, a common set of items was presented to children. Second, children’s results on the first stage determined the next set of more or less challenging questions that were presented to the child. Because not all children received the same questions, item response theory (IRT) scores were calculated to generate comparable scores across children and to facilitate longitudinal analysis of science achievement. The IRT scores estimate children’s performance on the science assessment as if they had been administered all items on the whole set of assessment questions (Najarian et al. 2009). The theta reliabilities for the measure for third, fifth, and eighth grades were all high: 0.88, 0.87, and 0.84 respectively (Najarian et al. 2009).

#### Socioeconomic status

SES was measured using income and parental education. Household income was measured based on parent reports and is expressed as the average value across all waves from kindergarten to the outcome grade, in units of 10,000 US dollars. The natural logarithm of this value was included in the models because prior studies have shown that income differences matter more for the achievement skills of children from more disadvantaged families (Votruba-Drzal 2006). The highest level of parental education was coded using a series of dummy variables that indicated whether the highest education was less than high school degree (reference group), high school, some college, bachelor degree, or graduate degree. Since education changes over time, it was coded cumulatively at each of third, fifth, and eighth grade to reflect the education level into which the parents were categorized for the majority of time across all available waves of data. Parents not having a majority of time in any of the levels were coded as having the highest reported educational level.

#### Child characteristics

Several characteristics of children were included in the analysis. Child age at the time of each outcome assessment was measured in months. Gender was included as a dummy variable (female reference group). Child race/ethnicity was represented with dummy variables indicating whether the child was White (reference group), Black, Hispanic, Asian, or from another race.

#### Parental and household characteristics

Marital status was entered as a dummy variable for being consistently married (not consistently married is reference group). Similarly to parental education, it was coded cumulatively at third, fifth, and eighth grade to reflect the category into which the parents were categorized for the majority of time across all available waves of data. Urbanicity was coded as a series of dummy variables for indicating if the children lived in communities that were rural (reference group), small town, sub-urban, small urban, or large urban (for a more detailed description see Miller et al. 2013).

These time-variant parental and household characteristics were created by aggregating all the values from kindergarten to the grade of the outcome. For the categorical variables, the most constant category was assigned. The families not having a majority of time in any of the categories were coded as having the most recent.

Finally, indicators of the opportunities for science learning children had with their family were created for the analysis. Two different indicators were created based on parents’ answers to two sets of questions regarding the frequency of promoting learning science inside of the home or outside of the home, which have previously shown to be separate factors with different effects on science outcomes (Lin and Schunn 2016). Opportunities for science learning in the home environment were measured using parent responses to two items asking parents how frequently in a typical week they talked about nature or did science projects with the children and helped children with science homework. A measure of opportunities for science learning out of the home environment was generated from questions asking the parent whether (1) since the beginning of the academic year, they attended a school event, such as a science fair, with their child; (2) whether in the past month, they visited a zoo, aquarium, or petting farm; (3) if they have visited a state or national park with their child; (4) whether their child attended a day or overnight camps; and (5) whether they visited an art gallery, museum, or historical site over the last summer. Although this last question asked about art or science museums, it was included as an opportunity for science learning given that children are noticeably more likely to go to science museums and natural history museums than to art museums (for a review, see Strager and Astrup 2014). The opportunities for learning variables were made by creating z-scores for parent responses on each item and predicting a factor based on regression scores. Then, factors across waves were averaged up to the predicted grade. Note that the indicators for the activities in the home were coded cumulatively up to third grade given that this set of questions were only included in the kindergarten, first grade, and third grade questionnaires, under the assumption that between-family relative levels of such behaviors would remain roughly stable in later years based on family routines established across 4 years.

#### School characteristics

There were two indicators of education quality regarding teachers: A continuous variable for the number of years of experience in teaching, and a dummy variable for indicating whether the science teacher has college training in science. Having training in science was coded differently depending on the grade: for third grade, teachers indicated whether they had science classes in college; for fifth and eighth grade, teachers indicated whether they received a degree in a science field. Also, a variable was created for the average number of hours of science instruction per week, aggregated across waves (i.e., amount of science instruction thus far) from kindergarten to eighth grade. This variable was derived from a questionnaire that was administered to science teachers asking them to report the number of hours per day and number of days per week that students received science. Finally, the type of school was entered as dummy variables to indicate whether the school is public (reference group), Catholic, other religious, or private (non-religious), which may capture the extent to which science instruction was influenced by national or state standards since only public schools are directly held accountable to such standards.

#### Reading and math achievement

Reading and math achievement were assessed using direct assessments similar in form to the science achievement test. Specifically, individualized measures were designed by the ECLS-K team to measure reading and math skills, and have been used in a number of research studies (Chatterji 2006; Claessens et al. 2009; Lubienski et al. 2013). The reading assessment measured literacy skills and reading comprehension. The mathematics assessment measured conceptual knowledge, procedural knowledge, and problem solving. Like in the science test, the reading and math assessments were given using a two-stage procedure and IRT scores were calculated for facilitating the longitudinal analysis of achievement. The theta reliability for reading at first, third, and fifth grade is 0.96, 0.94, and 0.93 respectively; whereas for math, it is 0.94, 0.95, and 0.95 for each grade (Najarian et al. 2009).

### Data analysis

Multilevel structural equation modeling (MSEM) was used to explore SES gaps in science achievement in elementary and middle school. MSEM combines the capabilities of multilevel models with those of structural equation modeling (SEM; Heck and Thomas 2015). This method can accommodate hierarchical data, with nesting of students (level 1) within schools (level two) by incorporating random effects for schools. Also, allows for the estimation of complex models of mediation that involve estimating multiple indirect effects simultaneously. Given that our model contains a random effect, the traditional fit indices are not applicable and are not presented. Additionally, in order to examine whether there are collinearity problems in the data and given that Mplus does not provide VIF statistics, we ran our models in a regression framework and found that all VIF statistics were in the acceptable range.

All models were estimated in Mplus (version 8.0; Muthén and Muthén 1998-2017), using a robust maximum likelihood estimator (MLR) and the TYPE=TWOLEVEL command. The models included sample weights in order to make the sample nationally representative.

**Fig. 1:**
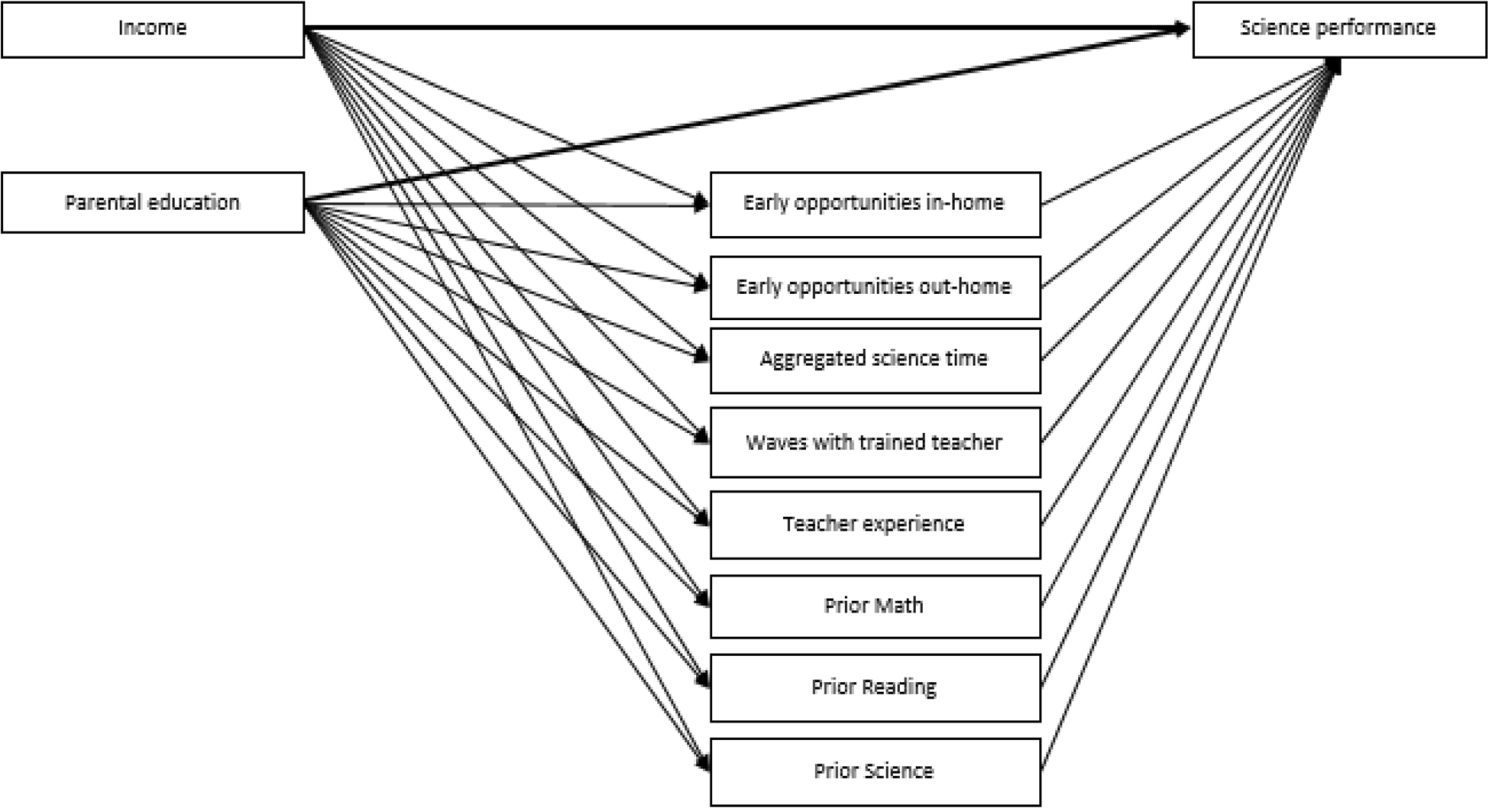
Diagram of mediation model estimated

The first aim of this study was to characterize the magnitude of disparities in science achievement related to household income and parental education. By including direct paths from SES to achievement, two unadjusted models were estimated in third, fifth, and eighth grades, one with household income and a second with parental education. These models characterized the magnitude and stability in SES gaps as children moved across grades. Next, direct paths from children, household, and school characteristics to science achievement were added to consider whether these variables account for the raw SES gaps found in the first step. The time-variant household characteristics were aggregated across the waves from kindergarten to eighth grade, and the models were estimated at each grade to examine how SES gaps in science changed over time. In the next step, early science stimulation variables were included to consider whether SES gaps in science achievement are explained by differences in early learning opportunities related to family and school contexts. In order to test for mediation effects, the last model included indirect paths from SES to science outcomes operating through the predictors of achievement: early learning opportunities for science, time of science class, waves with a science-trained, and teacher experience. In the final model, reading and math achievement scores from the prior assessment wave were introduced to the model to consider whether these skills mediate the relation between SES and science achievement skills. As a conservative approach, prior science scores are included here as well to get a purer measure of the effect of reading and math on science since all three are correlated for many reasons. A diagram of the full model tested in the last step is presented in Fig. 1.

## Results

Descriptive statistics for all variables in this study are shown in Table 1. The means and percentages are stable across grades, except those logically expected to change: children age, mean income is higher in eighth grade, parents are slightly more likely to have advanced degrees by eighth grade, students have more science instruction and more highly trained teachers in later grades, and all IRT scores increase by large amounts across grades. Tables 6, 7, and 8 in Appendix contain the correlations among the variables included in each of the models. Similar to prior work (Davis-Kean 2005), education and income were found to be only moderately correlated in each wave (around 0.03–0.04).

**Table 1:** Descriptive statistics for all variables

	Third grade	Fifth grade	Eighth grade
	M (S.D.) or %	M (S.D.) or %	M (S.D.) or %
SES			
Income ($10,000 units)	6.1 (4.9)	6.2 (4.9)	8.0 (6.2)
Parental education			
Less than high school	8%	7%	7%
High school	20%	19%	17%
Some college	33%	34%	33%
Bachelor’s degree	24%	24%	25%
Graduate degree	15%	16%	18%
Child			
Female	50%	50%	50%
Age (months)	111.0 (4.4)	134.6 (4.5)	171.3 (4.4)
Race			
White	62%	62%	62%
Black	10%	10%	10%
Hispanic	17%	17%	17%
Asian	6%	6%	6%
Other races	5%	5%	5%
Family			
Married	77%	76%	76%
Urbanicity			
Rural	16%	15%	15%
Town	13%	14%	13%
Suburban	35%	35%	35%
Small urban	20%	20%	20%
Large urban	16%	16%	17%
In-home science opportunities	0.009 (0.78)	0.009 (0.78)	0.009 (0.78)
Out-of-home science opportunities	0.002 (0.76)	0.017 (0.68)	0.013 (0.60)
School			
Type of school			
Public	79%	80%	80%
Catholic	13%	13%	13%
Other religious	6%	5%	5%
Other private	2%	1%	2%
Science class			
Years teaching	14.78 (6.68)	14.74 (5.94)	14.51 (5.33)
Teacher science training	94%	78%	77%
Science time per week	1.38 (0.80)	1.66 (0.80)	1.99 (0.81)
Academic achievement (IRT)			
Science	35.06 (9.93)	58.90 (14.22)	84.74 (16.05)
Math	44.30 (8.96)	86.17 (17.63)	115.28 (21.09)
Reading	56.81 (13.61)	109.09 (20.21)	140.91 (23.09)

### Unadjusted differences

Unadjusted differences in science achievement in third, fifth, and eighth grades are presented in the first column of Tables 2, 3, and 4 respectively. Disparities related to family income are shown in model 1 and gaps associated with parental education are displayed in model 2 (in both cases unadjusted for correlated family differences).

**Table 2:** Results for science at third grade

	Model 1		Model 2		Model 3		Model 4		Model 5	
	Coeff.	S.E.	Coeff.	S.E.	Coeff.	S.E.	Coeff.	S.E.	Coeff.	S.E.
Income	0.24***	0.02			0.12***	0.02	0.10***	0.02	0.04*	0.02
High school			0.13	0.09	0.10	0.09	0.11	0.09	0.01	0.02
Some college			0.27***	0.03	0.21***	0.03	0.21***	0.03	0.01	0.02
Bachelor			0.36***	0.03	0.27***	0.03	0.27***	0.03	0.04+	0.02
Graduate			0.37***	0.02	0.27***	0.02	0.26***	0.03	0.05*	0.02
Gender					0.14***	0.02	0.14***	0.01	0.11***	0.01
Age					0.10***	0.01	0.09***	0.01	− 0.03**	0.01
Black					− 0.13***	0.01	− 0.12***	0.02	− 0.05***	0.01
Hispanic					− 0.05***	0.01	− 0.05***	0.02	− 0.02+	0.01
Asian					0.01	0.03	0.01	0.01	0.01	0.01
Other races					− 0.01	0.02	− 0.01	0.02	− 0.02	0.01
No English					− 0.07***	0.05	− 0.06*	0.02	− 0.03**	0.01
Married					0.01	0.02	0.01	0.02	0.02*	0.01
Town					0.03+	0.02	− 0.01	0.02	0.01	0.01
Sub urban					0.02	0.04	− 0.01	0.03	0.00	0.02
Small urban					0.08*	0.03	0.02	0.02	0.03	0.02
Large Urban					0.02	0.04	− 0.01	0.01	0.00	0.01
Catholic					− 0.10***	0.02	− 0.01	0.01	− 0.03*	0.01
Other Relig.					− 0.06*	0.02	− 0.02	0.2	− 0.02+	0.01
Other Priv.					− 0.01	0.02	0.02*	0.01	0.02**	0.01
Opp. in home							0.06***	0.01	0.04***	0.01
Opp. out home							0.05**	0.01	0.02*	0.01
Years teaching							0.01	0.01	0.01	0.01
Trained teacher							0.01	0.01	0.01	0.01
Science time							0.01	0.02	0.02+	0.01
1st grade Math									0.16***	0.01
1st grade Reading									0.15***	0.01
1st grade Science									0.60***	0.01
Intercept	3.88***	0.15	3.29***	0.05	− 6.49***	1.80	− 4.62 *	2.35	− 2.62	2.40
Random										
Residual variances	0.94***	0.01	0.93***	0.01	0.86***	0.01	0.87***	0.01	0.43***	0.01
R-square	0.06		0.06		0.13		0.31		0.57	

***p < 0.001; **p < 0.01; *p < 0.05, +*p* < 0.10. Coeff = standardized coefficient

**Table 3:** Results for science at fifth grade

	Model 1		Model 2		Model 3		Model 4		Model 5	
	Coeff.	S.E.	Coeff.	S.E.	Coeff.	S.E.	Coeff.	S.E.	Coeff.	S.E.
Income	0.24***	0.02			0.11***	0.02	0.09***	0.02	0.06	0.09
High school			0.16*	0.07	0.13***	0.08	0.12***	0.03	0.01	0.02
Some college			0.34***	0.03	0.27***	0.03	0.27*	0.08	0.05+	0.02
Bachelor			0.41***	0.03	0.31***	0.03	0.31*	0.08	0.07**	0.02
Graduate			0.40***	0.03	0.30***	0.03	0.30**	0.11	0.06***	0.02
Gender					0.14***	0.01	0.13***	0.01	0.05***	0.01
Age					0.05***	0.01	0.05**	0.01	− 0.03**	0.01
Black					− 0.13***	0.02	− 0.12***	0.07	− 0.03**	0.02
Hispanic					− 0.02**	0.01	− 0.02**	0.07	− 0.01	0.02
Asian					0.02	0.04	0.03	0.04	− 0.02	0.01
Other races					− 0.01	0.01	− 0.01	0.03	− 0.02	0.01
No English					− 0.06*	0.07	− 0.04**	0.01	0.03	0.02
Married					0.01	0.08	0.14+	0.08	0.01	0.02
Town					− 0.01	0.02	− 0.01	0.02	− 0.02+	0.01
Sub urban					0.04	0.04	0.03	0.04	− 0.02	0.02
Small urban					0.03	0.03	0.03	0.02	− 0.03	0.01
Large urban					0.03	0.05	− 0.03	0.02	− 0.03	0.02
Catholic					− 0.06**	0.02	− 0.03	0.03	0.01	0.01
Other Rel.					− 0.05*	0.02	0.00	0.02	0.01	0.01
Other Priv.					0.02+	0.01	0.02*	0.01	0.02*	0.01
Opp. in home							0.04**	0.05	0.04	0.01
Opp. out home							0.04*	0.05	0.02	0.01
Years teaching							0.00	0.03	0.01	0.01
Trained teach							0.03	0.03	0.00	0.01
Science Time							0.04**	0.02	0.02*	0.01
3rd grade Math									0.17***	0.02
3rd grade Reading									0.16***	0.04
Science									0.56***	0.03
Intercept	4.75***	0.19	4.11***	0.36	− 2.64	2.60	3.41	2.60	0.30	0.84
Random										
Residual variances	0.94***	0.01	0.93***	0.01	0.89***	0.01	0.90***	0.01	0.88***	0.01
R-square	0.06		0.07		0.11		0.30		0.67	

***p < 0.001; **p < 0.01; *p < 0.05; +*p* < 0.10. Coeff = standardized coefficient

**Table 4:** Results for science at eighth grade

	Model 1		Model 2		Model 3		Model 4		Model 5	
	Coeff.	S.E	Coeff.	S.E	Coeff.	S.E	Coeff.	S.E.	Coeff.	S.E.
Income	0.23***	0.01			0.14***	0.02	0.10***	0.02	0.03+	0.02
High school			0.04	0.04	0.08	0.09	0.07	0.08	0.03	0.02
Some college			0.31***	0.06	0.26***	0.03	0.25***	0.03	0.03	0.02
Bachelor			0.57***	0.06	0.30***	0.03	0.30***	0.03	0.06	0.04
Graduate			0.59***	0.07	0.29***	0.03	0.28***	0.03	0.04*	0.02
Gender					0.10***	0.01	0.08***	0.02	0.05***	0.01
Age					0.01	0.01	0.02	0.01	− 0.03	0.03
Black					− 0.12***	0.02	− 0.12***	0.02	− 0.07***	0.02
Hispanic					− 0.03**	0.06	− 0.03**	0.02	− 0.04*	0.01
Asian					0.03*	0.01	0.02**	0.01	− 0.01	0.01
Other races					− 0.02	0.03	− 0.05***	0.01	− 0.03*	0.01
No English					− 0.04*	0.01	− 0.02	0.02	0.02	0.01
Married					0.01	0.08	0.02	0.02	0.01	0.01
Town					− 0.01	0.02	− 0.01	0.02	− 0.01	0.02
Sub urban					− 0.03	0.00	0.02	0.02	0.01	0.02
Small urban					0.03	− 0.01	0.01	0.02	0.00	0.02
Large urban					0.01	− 0.01	− 0.00	0.02	− 0.01	0.02
Catholic					− 0.05*	0.02	0.00	0.01	0.01	0.01
Other Rel					− 0.04+	0.02	0.01	0.01	0.00	0.01
Other Priv					0.01	0.02	0.02*	0.01	0.01	0.01
Opp. in home							0.01	0.02	0.01	0.01
Opp. out home							0.04*	0.02	0.01	0.01
Years teaching							0.02+	0.02	− 0.01	0.01
Trained teach							0.02	0.02	− 0.00	0.02
Science time							0.04*	0.02	0.03	0.03
5th grade Math									0.15***	0.02
5th grade Reading									0.12***	0.03
5th grade Science									0.61***	0.02
Intercept	6.16***	0.25	6.48***	0.42	− 1.09	0.57	− 2.01	4.16	1.30***	0.29
Random										
Residual variances	0.95***	0.01	0.94***	0.01	0.91***	0.01	0.92***	0.01	0.91***	0.01
R-square	0.05		0.06		0.09		0.29		0.69	

***p < 0.001; **p < 0.01; *p < 0.05; +*p* < 0.10. Coeff = standardized coefficient

When it comes to family income, there are significant associations between family income and science achievement that grow slightly over time. More specifically, a unit increase in the natural log of income is linked to 0.24 SD, 0.24 SD, and 0.23 SD unit increases in science achievement skills across third, fifth, and eighth grades. It can be seen that science achievement gaps between children from low- and middle-income families are perceivable but stable from third to eighth grade.

Associations between parental education and science skills are significant as well, as shown in model 2. Students whose parents have less than a high school degree serve as the reference group. These gaps are virtually unchanged as children move through grades. In particular, the gaps between children with the least educated parents and those whose parents had a high school degree were 0.11 SD, 0.16 SD, and 0.04 SD, although only was significative for the fifth grade. Having a parent with some college was linked to 0.27 SD higher science achievement than children whose parents have less than a high school education. Gaps between children whose parents did not graduate from high school and children whose parents had a bachelor or graduate degree were quite sizable and hovered around 0.36 SD and 0.37 SD respectively. In general, associations between family income and parental education with science achievement were stable across third, fifth, and eighth grades.

#### Child and family demographics

In model 3 of Tables 2, 3, and 4, demographic factors were added as predictors with both income and parental education, to examine whether science achievement gaps related to income and education attenuated after taking into account these closely associated factors, and to isolate income from parental education effects.

The income gaps in science achievement declined after the demographic characteristics were entered, such that the income coefficients dropped by 50% at third grade, 42% at fifth grade, and 40% at eighth grade. However, the effects of income were still large and statistically significant; parental income *per se* (rather than its associations through other demographic factors) appears to influence student achievement in science, although the magnitude of the coefficient drops in half.

Disparities related to parental education also attenuated but still were statistically significant. Education gaps were reduced between 16 and 49%, with greater reductions at higher levels of education. Despite these reductions, parental education was still associated with large increases in science achievement. Thus, like with income effects, there is evidence that a large fraction of the association of parental education with achievement stems from income and race/ethnic differences but meaningful differences related to parental education remains.

Several child and family characteristics emerged as consistent predictors of science achievement, including race/ethnicity, language at home, gender, and age. Across all three grades, children of Black and Hispanic backgrounds tended to score below White students. Asian students did not differ from White students at third and fifth grades, but obtained higher scores in eighth grade. Children with no English at home performed lower across all grades. In third and fifth grades, older students outscored their younger counterparts, and across all waves, boys tended to score higher than girls. Also, in third grade, children living in small-urban communities outscored their rural peers. Across all waves, when compared to students in public schools, students attending catholic and religious schools scored lower. In other words, most of the potential confounds with parental education and family income (i.e., ethnicity, home language, urbanicity, and school type) were in fact contributing to the raw education and income gaps, highlighting the importance of disaggregating the gaps in this way.

#### School characteristics and science experiences

School characteristics and opportunities for science learning at home and in school were introduced in model 4 of Tables 2, 3, and 4. Overall, these new factors slightly reduced links between SES and science achievement in third, fifth, and eighth grades; much less reduction than the demographic factors introduced previously. In particular, the income and education coefficients typically fell by 0.01–0.02 SD.

Several of the factors introduced in model 4 were significantly linked with science achievement. More frequent opportunities for science learning in the home environment were associated with science achievement at third and fifth grade, and learning opportunities outside of the home environment related to higher levels of science achievement skills across all waves. In third grade, teacher’s overall experience and having a trained teacher were only marginally associated with science performance. In fifth and eighth grade, a new positive association emerged between the amount of science instruction and science achievement.

#### Prior academic skills

Children’s science, reading, and math skills from the prior assessment wave were introduced in model 5 of Tables 2, 3, and 4 to examine whether SES gaps in science skills are explained by differences in reading and math skills when controlling by previous science performance. In fact, in third, fifth, and eighth grades differences in science, reading, and math skills explain a large proportion of remaining SES disparities in science achievement. When it came to economic gaps in science skills, reductions were 83%, 75%, and 87% in third, fifth, and eighth grades respectively. As a result, the association between income and science was no longer significant in fifth and eighth grades, and small but still statistically significant in third grade after taking into account science, reading, and math skills. Science disparities linked to parental education were not significant, with the exception of graduate education. Graduate education remained significant but dropped 87% in third grade, and 93% in eighth grade. Associations between education and science achievement remained statistically significant in fifth grade after introducing measures of prior science, reading, and math skills into the models, although the magnitude of the associations between parental education and science skills dropped. Having parents with high school degree was not statistically different than having parents without high school in any grade. Some college dropped 86%, having bachelor education dropped 82%, and having graduate education dropped 85%. Thus, differences in prior science, reading, and math skills seem to also be an important pathway through which parental education shapes science skills.

Overall, when compared to the unadjusted differences in model 1, income coefficients in the fully adjusted model 5 fell by between 75 and 87%. Similarly, unadjusted differences related to education shown in model 2 fell almost entirely at third grade, and between 85 and 93% in fifth and eighth grades. Thus, the variables examined here explain a large portion of the unadjusted SES gaps in science achievement.

#### Mediation analysis

**Fig. 2:**
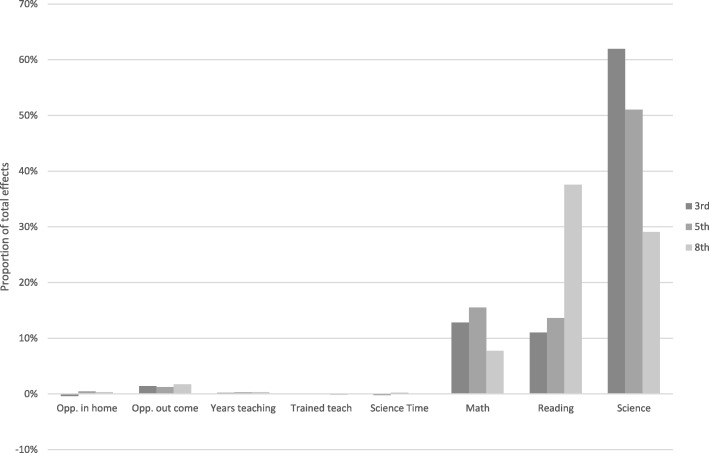
Contributions of each mediator to the income gap in science achievement

**Fig. 3:**
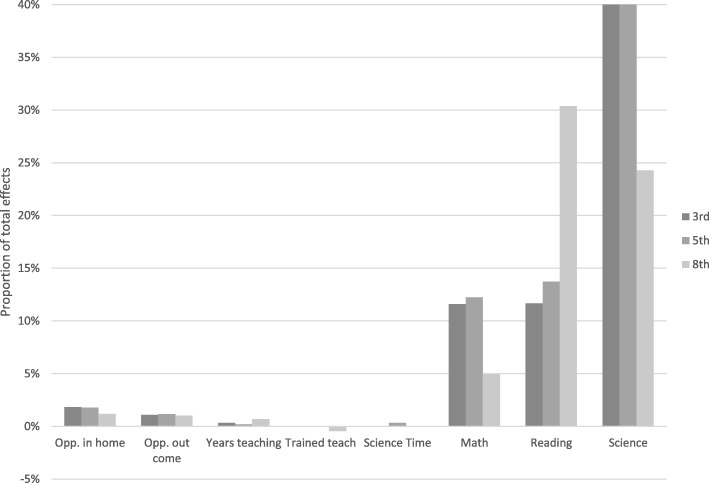
Contributions of each mediator to the education gap (Bachelor’s vs. no high school) in science achievement.

Table 5 displays the result of the formal tests of mediation that were performed to examine which parts of SES gaps were operating indirectly through early opportunities for science learning in and outside of the home environment, science instructional time, science specific teacher training, and prior science, reading, and math skills. Previously presented analyses were estimated simultaneously with the mediations in order to identify which variables account for which gaps. Mediation allows for the identification of, for example, whether science instructional time is connected to household income and therefore potentially part of the income gap in science. As a summary, Figs. 2 and 3 present the relative amount each factor contributes to the income and education gaps at each grade level.

**Table 5:** Mediation of science inputs between SES and science achievement (from model 5)

	In home opp.		Out of home opp.		Science time		Trained teacher		Years teaching		Prior Math		Prior Reading		Prior Science	
	Coeff.	S.E.	Coeff	S.E.	Coeff.	S.E.	Coeff.	S.E.	Coeff	S.E.	Coeff	S.E.	Coeff	S.E.	Coeff	S.E.
3rd grade																
Income	− 0.03	0.02	0.21***	0.02	− 0.03	0.03	-0.01	0.03	0.06**	0.03	0.24***	0.02	0.22***	0.02	0.31***	0.02
HighSch	0.11***	0.03	0.03	0.03	0.00	0.03	0.05	0.03	0.14***	0.03	0.08*	0.02	0.13***	0.03	0.20***	0.03
SomColl	0.18**	0.04	0.16***	0.04	− 0.02	0.04	0.01	0.04	0.12***	0.03	0.20***	0.03	0.23***	0.04	0.34***	0.04
Bachelor	0.17**	0.04	0.20***	0.04	− 0.01	0.04	0.01	0.04	0.12***	0.03	0.27***	0.04	0.29***	0.03	0.39***	0.04
Graduate	0.20***	0.03	0.23***	0.03	0.01	0.03	0.05+	0.03	0.09**	0.03	0.25***	0.03	0.30***	0.02	0.35***	0.03
5th grade																
Income	0.04+	0.02	0.21***	0.02	0.03	0.03	0.04	0.03	0.10***	0.02	0.31***	0.02	0.29***	0.02	0.31***	0.02
HighSch	0.10*	0.03	0.06*	0.03	0.05+	0.03	0.05	0.03	0.08*	0.03	0.10***	0.03	0.12***	0.03	0.15***	0.03
SomColl	0.17***	0.04	0.18***	0.03	0.04	0.03	0.07	0.04	0.10*	0.04	0.20***	0.03	0.23***	0.04	0.27***	0.03
Bachelor	0.16***	0.03	0.21***	0.03	0.06*	0.03	0.07*	0.04	0.07*	0.03	0.26***	0.03	0.31***	0.03	0.33***	0.03
Graduate	0.20***	0.03	0.24***	0.03	0.08**	0.03	0.06*	0.03	0.10**	0.03	0.25***	0.03	0.29***	0.03	0.32***	0.08
8th grade																
Income	0.04	0.03	0.22***	0.02	-0.03	0.03	0.02	0.02	0.04*	0.02	0.33***	0.02	0.32***	0.02	0.31***	0.02
HighSch	0.14**	0.04	− 0.01	0.03	0.01	0.03	0.03	0.04	0.09**	0.03	0.08**	0.03	0.12***	0.03	0.12***	0.03
SomColl	0.21***	0.04	0.08*	0.04	0.02	0.04	0.06	0.04	0.12**	0.04	0.18***	0.03	0.25***	0.04	0.26***	0.03
Bachelor	0.19***	0.03	0.16***	0.04	0.01	0.04	0.07*	0.04	0.11**	0.04	0.26***	0.03	0.32***	0.03	0.32***	0.03
Graduate	0.26***	0.03	0.18***	0.04	0.03	0.04	0.04	0.04	0.14***	0.04	0.24***	0.03	0.31***	0.03	0.30***	0.08

***p < 0.001; **p < 0.01; *p < 0.05; +*p* < 0.10. Coeff = standardized coefficient

The mediation effects for income were essentially the same across all three grades: income shaped science achievement through math and reading differences (the indirect effects ranged between 0.34 and 0.86, *p* < 0.001) and out of home opportunities at third (0.04, *p* < 0.05) Specifically, when compared to children from low-income families, children from middle- and upper-income families scored much higher on reading and math skills, which lead to large enhancements in science achievement. The contribution of out-of-home learning indicated the greater number of science learning opportunities provided by higher income families benefited their children’s science achievement. For the mediation effects of parental education, a number of indirect effects were similar to the mediation results for income (see Table 5). In particular, parental education indirect effects were also strongly connected to prior math and reading (ranging from 0.016 to 0.067, *p* < 0.001) and to out of home opportunities at third grade.

Interesting, there were consistent effects of in home opportunities at all grade levels: more highly educated parents provided additional opportunities for science learning in the home environment. However, the indirect effect was only significant for learning at third grade. Having some college (0.007, *p* < 0.01), having a bachelor degree (0.008, *p* < 0.01), and having graduate education (0.08, *p* < 0.01) was associated with larger science scores. This difference of informal science is intuitive: income alone can be used to purchase out-of-home experiences, but education seems more central for the provision of enrichment in the home environment. It is important to note that there were almost no significant indirect effects through science instructional time in school. The only exception was that having a parent with a bachelor or graduate degree at fifth grade was associated with greater amounts of science instructional time (0.003, *p* < 0.05).

Although there was the largest variability in science instructional time in fifth grade and amount of science instructional time predicted greater science achievement, neither income nor parental education were associated with science instructional time and thus this was not a pathway for income or education gaps. This stands in contrast to the lack of a mediation effect via teacher training: for the measure of teacher training used here, there was no relationship to achievement outcomes. The main differences across grade levels relate to the relative magnitude of effects for parental education. For third grade, more of the achievement gap is related to in the home and out of the home informal learning opportunities, whereas for fifth and eighth grade, more of the effect comes from prior math, reading, and science.

### General discussion

The central goal of this investigation was to characterize and explain socioeconomic gaps in science achievement in elementary and middle school related to parental income and education. Given the need for STEM-related professionals in the US and other countries and the widening of the SES achievement gaps, it is important to describe the early emergence and development of science gaps. This study provides crucial information for programs and policies interested in enhancing access to high educational attainment and careers in science for children from families across the SES distribution. Our results show that in the U.S., similar to early gaps in mathematics and reading achievement (Galindo and Sonnenschein 2015; Nores and Barnett 2014; National Research Council 2009; Wang et al. 2013), there are large gaps associated with parental income and parental education, even when controlling for each other and also the correlated and frequently implicated factors of race/ethnicity (Muller et al. 2001; Quinn and Cooc 2015), family structure, language at home, and urbanicity (Miller et al. 2013; Votruba-Drzal et al. 2016). Controlling for correlated factors is important because the simple associations of achievement with income alone or parental education alone are twice as large as the estimates obtained once these correlated factors are considered simultaneously. For research and policy, it is now clear that separate consideration must be given to understanding the influences of parental income, parental education, and ethnicity on student achievement. Large effects were apparent as early as 3^rd^ grade and remained similarly large through 5^th^ and 8^th^ grade.

Higher levels of parental education (i.e., some college, high school, bachelor’s, graduate school) predicted significant increases in science achievement. Interestingly, the primary pathway by which parental education influenced science achievement was through mathematics and reading achievement. This particular pathway would explain why there were large parental education effects even in the early grades where presumably providing children with science learning experiences does not depend upon graduate level or even undergraduate level knowledge of science. Further, this pathway increased in strength across grades. However, there were also effects related to the kinds of science experiences provided in and outside of the home, which when combined are substantial in size.

Income *per se* (i.e., not parental education and not ethnicity or urbanity or the host of other demographic factors) was also associated with early science achievement. Prior literature has discussed the role of income in terms of (1) indirect indicators of parental education (Guryan et al. 2008; Ramey and Ramey 2010), (2) providing access to informal science learning opportunities (Bradley et al. 2001; Lee 2009), or (3) providing access to higher quality education (Duncan and Brooks-Gunn 1997; Ferguson et al. 2007). Although we found support for the first two explanations, we also showed that income mostly relates to science through its associations with mathematics and reading achievement, which likely involve access to higher quality education in general, just not effects specific to science instruction.

Interestingly, the parental income effects were not mediated by science class time effects and the parental education effects were mediated by class time only at the highest educational levels at fifth grade. It may be that more localized school factors of principal interests, state or school district mandates, or teacher interests drive the relative amounts science time and these factors are not correlated with parental education or income.

### Caveats

The current analyses were fundamentally correlational in nature and thus cannot definitively establish causality of the parental income and education gaps. At the same time, the inclusion of variables that are likely sources of third variable confounds and the examination of longitudinal data that carefully separates inputs from outputs does increase confidence about the likely causal nature of the observed effects. Further, intervention studies on reading strategies with struggling readers have found to lead to improved science outcomes (Jackson and Ash 2011; Seifert and Espin 2012), and thus some elements of the overall model have already been shown to be causal.

The examination of pathways that explain SES disparities in science achievement is incomplete. Gaps related to parental education and income were still significant in the final model, with the exception that education effects in third grade and income effects in fifth and eighth grade were no longer significant. These unexplained effects accounted for 6–8% of the gaps in third and eighth grades, and for 6–30% of the gaps in fifth grade. It could be that better measures of the factors that were examined could account for even more of the variance (e.g., other measures of teaching quality such as rigor of the science that is taught, measures of more kinds of informal science exposure such as frequency of conversations about science topics or amount of science-related reading, or measures of the reading and mathematics skills most relevant to science such as quality of inferences about non-fiction texts or ability to decode tables and graphs). Alternatively, it could be that other factors matter. Most saliently, it was surprising that teacher training and amount of experience were not drivers of the parental income and education gaps. Those variables are often associated with achievement gaps since the students with the highest needs often are taught by teachers with the weakest preparation (Muijs et al. 2004; Nasir et al. 2011). Future studies should examine other indicators of teacher quality. Yet, it is also important to note that the current analyses have uncovered the largest sources of the income and education gaps.

This study examined science achievement using one holistic measure that did not attempt to decouple effects separately by disciplines within science (e.g., biology which may be more driven by early experiences in nature that are harder to obtain in cities or physics which may be more driven by mathematics). This measure did not also decouple understanding of cross-cutting concepts from core content within disciplines, ability to engage in the practices of science, or attitudes about the nature of science. For example, it could be that some of the effects of either parental income or education are larger for core content given the central role of reading. Future studies will have to decompose the science achievement effects along these lines.

This examined dataset also treated parental education and income purely in terms of levels, ignoring potential variations in parental education and occupations that likely also have important effects on science achievement. For example, whether the parents studied science vs. other topics in university or have science or engineering-related job clearly may lead to additional effects on student learning in science, either by providing homework help or setting higher value or expectations for achievement in science. Since science-related jobs are relatively high paying (Lacey and Wright 2009) and typically require at least bachelors and often graduate degrees, future studies of SES achievement gaps in science should also explore the mediating role of parental occupation.

The current study was also focused on outcomes in the USA, which creates potential limitations to generalizability to other countries. However, the USA is diverse along many dimensions of relevance to science education, with some states performing at similar levels to the highest performing countries in the PISA and TIMSS cross-national comparisons and other states performing at similar levels to the lowest performing countries. Yet there is some evidence that the relationships of child characteristics like interest in science with science achievement is uneven across countries (Tucker-Drob et al. 2014), suggesting that some specificity to at least developed nations is likely.

## Conclusions

### Implications for research

Now that components of the SES effects have been established for early science achievement, and some differences of underlying pathways have been revealed, it is clear that future research should separately measure and analyze parental education, parental income, and ethnicity, rather than continuing to treat them as one holistic construct, as has typically been the case in the past. Further, as noted above, we recommend including parental occupation to help better uncover likely important variations.

Most saliently, the current studies now place greater attention on research that will uncover the nature and generality of the reading and mathematics effects, especially as new standards in mathematics, reading, and science call for more complex forms of reasoning in early mathematics, reading, and science instruction, as well as greater integration of reading and mathematics in science (Common Core State Standards Initiative 2010a, 2010b; NGSS Lead States 2013). More research is needed to understand how mathematics and reading influences learning in science, and whether it does so equally in all kinds of science learning environments. For example, instruction that increases the integration of reading and mathematics into science instruction may exacerbate parental income and education effects in science because of their large effects on mathematics and reading or such instructional change may mitigate the effects by providing greater opportunities for all students to improve underlying mathematics and reading abilities.

Another important question is whether mathematics and reading achievement shape science *learning* or if the science achievement test itself simply has a high reading load or involves mathematical calculations or deciphering graphs and tables. Such research could examine the separate effects of prior mathematics and reading achievement (which is influencing science learning) and concurrent mathematics and reading achievement (which is influencing test performance).

Finally, although ethnicity effects were not the focus in this study, important features of ethnicity effects were revealed by the current analyses that suggest important directions for future research. Looking across models 3 and 4, it can be seen that ethnicity effects are large and quite stable in size; that is, they are not fully explained by income and parental education effects and they are also not explained by urbanicity, school type, amount of science instruction, and teacher training. Most notably, gaps between Black and White students taken from model 3 of Tables 2, 3, and 4 were around 0.25 SD across grades. Science disparities between Hispanic and White students were between 0.06 SD and 0.04 SD. Similar to parental education and income gaps, the ethnicity gaps are substantially reduced when mathematics and reading achievement are included in the model, although the remaining unexplained gaps are still large, larger than the unexplained gaps for parental education and income.

### Implications for practice

Results of this research have important implications for the development of science skills during elementary and middle school, which in turn can affect the interest and performance in STEM fields across the life course. Overall, the central role of mathematics and reading achievement in accounting for the large and growing SES gaps in early science achievement suggest that policies that reduce the SES gaps in mathematics and reading are especially important. One construal of science education policy for the early grades pits science against the other content areas in a fight for instructional time; indeed, there is relatively little time spent on science instruction in the early grades, as shown by our analyses. However, our current findings suggest that elementary instructional approaches that simultaneously address science instruction with reading and/or mathematics instruction will likely be especially important for improving overall science outcomes (O’Reilly and McNamara 2007). For example, integrated reading/science curricula have been developed that improve both science learning and reading performance (e.g., Pearson et al. 2010). Access to such curricula for schools serving broadly low-performing students is likely to be especially important, whereas schools already high performing in reading would not need such approaches. Successful implementation of new curricula depends upon supports for teachers (Pareja Roblin et al. 2018). Our results show that low-income children (but not children of parents with lower education levels) are likely to have inexperienced teachers. It is likely that successful interventions will need to not only acquire reform reading/science curricula but also financial support for aligned teacher professional development.

Additionally, this study shows that SES disparities in science achievement emerge early and are well-established by third grade, thus programs and policies aimed at addressing these gaps may need to target children when they are young, perhaps even earlier during the early elementary and preschool years. One promising framework for engaging young learners is the Design Make and Play learning methodology which aims to increase children’s motivation and engagement in science learning by engaging children in meaningful activities in formal or informal settings that leverage children’s innate curiosity in understanding the world around them (Honey and Kanter 2013). Our results make clear that in-home interventions will tend to be differentially accessed as a function of parental education, but not parent income levels, whereas out-of-home interventions will be differentially access as a function of parent income levels and (to a smaller extent) parental education. Thus, financial subsidies will be important for out-of-home interventions, whereas information campaigns and parent-learning opportunities may be needed to allow these informal learning opportunities to address rather than increase SES gaps in science.

Beyond these sorts of efforts to explicitly engage children in science learning opportunities, the results of this study would suggest that efforts to expand access to public pre-K programs for 3 and 4 year olds, which are effective in attenuating socioeconomic gaps in reading and math achievement (Votruba-Drzal et al. 2013), may benefit science achievement in the longer term, since reading and math achievement emerged as the most salient pathways through which both income and parental education influenced science achievement. After school science programs may further address the lack-of-access problems associated with parental education and family income; however, information campaigns may be needed to ensure that children have equal likelihood of participating across parental education levels given that both income and education level were predictive.

Finally, the very large gaps of parental education and income on early science achievement show that the USA is currently far from achieving its ideals of equitable opportunities for science learning, and such a state of affairs is particularly problematic for a country that places science and engineering at such a central role for its future prosperity.

## Appendix

**Table 6:** Correlations among all third grade predictors

	White	Black	Hispa.	Asian	Other race	No English	Female	Age	Income	Mary	Below HS	High school	Some college	Bache	Graduate	Town	Sub urban	Small urban	Large urban	Catholic	Other religion	Other Priv.	Opp-in-home	Opp-out-home	Years teaching	Train teach	Science time	Previous Math
Black	− 0.46																											
Hispanic	− 0.57	− 0.11																										
Asian	− 0.31	− 0.06	− 0.08																									
Other races	− 0.34	− 0.07	− 0.08	− 0.05																								
NoEnHome	− 0.41	− 0.09	0.46	0.27	− 0.02																							
Female	0.02	− 0.01	− 0.01	− 0.02	− 0.02	− 0.01																						
Age	0.09	− 0.03	−0.04	− 0.07	− 0.03	− 0.06	0.09																					
Income	0.32	− 0.30	− 0.17	0.05	− 0.05	− 0.16	0.03	− 0.04																				
Married	0.21	− 0.30	− 0.03	0.05	− 0.05	0.01	0.03	0.00	0.46																			
Below HS	− 0.22	0.09	0.22	0.00	0.00	0.24	0.02	− 0.01	− 0.32	− 0.16																		
HighSch	− 0.07	0.09	0.03	− 0.04	0.01	0.00	0.01	0.01	− 0.31	− 0.16	− 0.05																	
SomColl	− 0.03	0.05	0.02	− 0.06	0.02	− 0.05	− 0.03	− 0.01	− 0.16	− 0.03	− 0.16	− 0.30																
Bache	0.10	− 0.08	− 0.07	0.05	− 0.03	− 0.04	0.00	0.01	0.26	0.13	− 0.13	− 0.27	− 0.43															
Graduate	0.11	− 0.10	− 0.10	0.07	− 0.01	− 0.03	0.01	− 0.01	0.40	0.16	− 0.10	− 0.21	− 0.33	− 0.27														
Town	0.06	− 0.03	− 0.09	− 0.03	0.09	− 0.10	0.01	0.05	− 0.07	− 0.01	− 0.03	0.03	0.03	− 0.02	− 0.03													
Sub urban	0.06	− 0.05	− 0.03	0.03	− 0.05	− 0.01	0.02	− 0.07	0.20	0.08	− 0.05	− 0.07	− 0.05	0.08	0.07	− 0.30												
Small urban	− 0.01	0.04	0.01	0.00	− 0.05	0.01	0.00	0.03	0.02	− 0.01	0.02	− 0.03	− 0.02	0.00	0.04	− 0.22	− 0.38											
Large urban	− 0.23	0.04	0.23	0.07	− 0.01	0.24	− 0.02	− 0.01	− 0.02	− 0.02	0.05	-0.03	− 0.02	0.00	0.02	− 0.16	− 0.28	− 0.20										
Catholic	0.07	− 0.09	− 0.01	0.01	− 0.03	− 0.02	0.01	0.03	0.18	0.09	− 0.08	− 0.10	− 0.05	0.12	0.08	0.03	− 0.09	0.10	0.13									
Other Rel	0.08	− 0.03	− 0.03	− 0.04	− 0.03	− 0.04	0.00	0.05	0.10	0.05	− 0.06	− 0.05	− 0.03	0.05	0.07	0.02	− 0.04	0.07	0.02	− 0.11								
Other Priv	0.00	− 0.03	− 0.02	0.04	0.03	− 0.02	− 0.01	− 0.02	0.17	0.01	− 0.03	− 0.06	− 0.07	0.01	0.15	− 0.04	0.00	0.06	0.04	− 0.06	− 0.04							
Opp-in-home	0.03	0.01	− 0.06	− 0.03	0.02	− 0.10	0.03	− 0.03	0.01	0.04	− 0.07	− 0.04	0.01	0.01	0.05	0.03	− 0.04	0.01	− 0.05	− 0.03	− 0.03	0.01						
Opp-out-home	0.11	− 0.10	− 0.04	0.01	− 0.04	− 0.06	0.01	0.00	0.29	0.10	− 0.15	− 0.17	− 0.05	0.09	0.21	− 0.06	0.09	0.03	0.04	0.07	0.05	0.11	0.09					
Years teaching	0.12	− 0.03	− 0.10	− 0.01	− 0.04	− 0.11	0.01	0.04	0.06	0.00	− 0.08	0.00	0.00	0.02	0.03	0.07	− 0.02	− 0.01	− 0.04	− 0.02	0.02	0.00	− 0.01	0.03				
Trained teacher	0.03	0.00	− 0.01	− 0.04	− 0.01	− 0.01	− 0.02	0.03	0.00	− 0.01	− 0.02	0.00	− 0.02	0.02	− 0.01	0.04	− 0.02	0.00	− 0.04	0.01	− 0.11	− 0.02	0.02	− 0.03	0.05			
Science time	− 0.03	0.09	− 0.02	0.01	− 0.04	− 0.03	− 0.01	0.00	− 0.04	− 0.05	− 0.01	0.03	0.00	− 0.01	0.00	0.00	0.01	0.00	− 0.04	− 0.10	− 0.09	0.04	0.02	0.01	− 0.04	0.02		
Previous Math	0.26	− 0.22	− 0.14	0.03	− 0.07	− 0.13	0.04	0.16	0.36	0.19	− 0.20	− 0.19	− 0.07	0.15	0.21	− 0.05	0.08	0.04	− 0.02	0.09	0.08	0.07	− 0.01	0.15	0.04	0.02	0.00	
Previous Read	0.18	− 0.15	− 0.13	0.08	− 0.05	− 0.13	− 0.10	0.08	0.35	0.19	− 0.20	− 0.18	− 0.06	0.13	0.21	− 0.04	0.08	0.04	0.00	0.10	0.10	0.07	0.01	0.14	0.05	0.01	− 0.01	0.66

**Table 7:** Correlations among all fifth grade predictors

	White	Black	Hispa.	Asian	Other race	No English	Female	Age	Income	Mary	Below HS	High school	Some college	Bache	Graduate	Town	Sub urban	Small urban	Large urban	Catholic	Other religion	Other Priv.	Opp-in-home	Opp-out-home	Years teaching	Train Teach	Science time	Previous Math
Black	− 0.44																											
Hispanic	− 0.58	− 0.13																										
Asian	− 0.29	− 0.06	− 0.08																									
Other races	− 0.32	− 0.07	− 0.09	− 0.05																								
NoEnHome	− 0.45	− 0.10	0.51	0.24	− 0.03																							
Female	0.00	0.00	0.00	− 0.01	− 0.02	0.00																						
Age	0.11	− 0.03	− 0.07	− 0.07	− 0.02	− 0.09	0.08																					
Income	0.37	-0.29	− 0.22	0.04	− 0.06	− 0.24	0.02	− 0.01																				
Married	0.20	− 0.29	− 0.02	0.05	− 0.04	0.01	0.02	0.01	0.44																			
Below HS	− 0.25	0.07	0.26	− 0.01	− 0.02	0.30	0.00	− 0.02	− 0.35	− 0.12																		
HighSch	− 0.08	0.09	0.04	− 0.04	0.01	0.03	0.01	0.00	− 0.32	− 0.17	− 0.11																	
SomColl	− 0.02	0.05	0.01	− 0.05	0.03	− 0.06	− 0.02	0.00	− 0.14	− 0.05	− 0.18	− 0.33																
Bache	0.11	− 0.08	− 0.09	0.04	− 0.01	− 0.07	0.00	0.01	0.27	0.13	− 0.14	− 0.27	− 0.42															
Graduate	0.13	− 0.10	− 0.10	0.06	− 0.03	− 0.06	0.02	0.00	0.40	0.16	− 0.11	− 0.21	− 0.33	− 0.27														
Town	0.07	− 0.03	− 0.10	− 0.02	0.09	− 0.10	0.01	0.05	− 0.06	− 0.01	− 0.05	0.04	0.04	− 0.02	− 0.03													
Sub urban	0.08	− 0.06	− 0.04	0.03	− 0.05	− 0.03	0.02	− 0.06	0.21	0.06	− 0.06	− 0.07	− 0.06	0.09	0.07	− 0.30												
Small urban	− 0.02	0.05	0.01	0.00	− 0.05	0.01	− 0.01	0.01	0.01	0.00	0.03	− 0.02	− 0.02	0.00	0.03	− 0.21	− 0.38											
Large urban	− 0.26	0.05	0.27	0.07	− 0.02	0.28	− 0.02	− 0.03	− 0.07	− 0.04	0.11	− 0.03	− 0.02	− 0.01	0.00	− 0.17	− 0.30	− 0.21										
Catholic	0.08	− 0.08	− 0.03	0.01	− 0.03	− 0.05	0.00	0.02	0.19	0.09	− 0.09	− 0.11	− 0.05	0.12	0.08	0.02	− 0.07	0.09	0.12									
Other Rel	0.08	− 0.03	− 0.04	− 0.03	− 0.03	− 0.05	0.00	0.03	0.11	0.05	− 0.06	− 0.06	− 0.03	0.05	0.08	0.02	− 0.04	0.07	0.00	− 0.10								
Other Priv	0.01	− 0.04	− 0.02	0.03	0.03	− 0.03	− 0.01	− 0.03	0.15	0.02	− 0.03	− 0.05	− 0.05	0.00	0.13	− 0.02	− 0.03	0.07	0.03	− 0.05	− 0.03							
Opp-in-home	0.05	0.01	− 0.08	− 0.03	0.03	− 0.12	0.03	− 0.01	0.03	0.03	− 0.09	− 0.04	0.00	0.02	0.07	0.04	− 0.04	0.01	− 0.06	− 0.02	− 0.03	0.01						
Opp-out-home	0.12	− 0.10	− 0.06	0.01	− 0.04	− 0.08	0.01	0.00	0.30	0.09	− 0.15	− 0.17	− 0.05	0.11	0.20	− 0.06	0.08	0.02	0.04	0.08	0.05	0.08	0.10					
Years teaching	0.18	− 0.06	− 0.15	0.00	− 0.03	− 0.14	0.00	0.06	0.12	0.03	− 0.11	− 0.03	0.01	0.03	0.06	0.08	− 0.01	0.01	− 0.09	− 0.02	0.04	0.02	0.01	0.05				
Trained teacher	0.06	− 0.02	− 0.05	− 0.03	0.02	− 0.07	0.00	0.05	0.02	0.00	− 0.03	0.00	0.00	0.01	0.00	0.05	− 0.03	0.03	− 0.06	− 0.04	− 0.06	0.00	0.04	− 0.01	0.01			
Science time	0.03	0.09	− 0.06	− 0.02	− 0.06	− 0.07	− 0.01	0.04	0.00	− 0.03	− 0.03	0.01	− 0.01	0.01	0.02	− 0.01	0.03	− 0.01	− 0.04	− 0.05	− 0.07	0.01	0.02	0.01	− 0.03	0.05		
Previous Math	0.28	− 0.24	− 0.15	0.05	− 0.07	− 0.14	0.11	0.11	0.41	0.21	− 0.22	− 0.19	− 0.08	0.16	0.23	− 0.04	0.10	0.04	− 0.04	0.05	0.07	0.06	0.02	0.17	0.09	0.03	0.01	
Previous Read	0.30	− 0.20	− 0.19	0.03	− 0.07	− 0.21	− 0.09	0.09	0.44	0.20	− 0.26	− 0.20	− 0.07	0.18	0.25	− 0.02	0.08	0.03	− 0.06	0.12	0.08	0.07	0.03	0.20	0.12	0.04	0.02	0.72

**Table 8:** Correlations among all eighth grade predictors

	White	Black	Hispa.	Asian	Other race	No English	Female	Age	Income	Mary	Below HS	High school	Some college	Bache	Graduate	Town	Sub urban	Small urban	Large urban	Catholic	Other religion	Other Priv.	Opp-in-home	Opp-out-home	Years Teaching	Train teach	Science time	Previous Math
Black	− 0.44																											
Hispanic	− 0.58	− 0.12																										
Asian	− 0.30	− 0.06	− 0.08																									
Other races	− 0.32	− 0.07	− 0.09	− 0.05																								
NoEnHome	− 0.45	− 0.10	0.52	0.25	− 0.03																							
Female	0.01	− 0.01	0.00	− 0.01	− 0.02	0.00																						
Age	0.10	− 0.01	− 0.06	− 0.08	− 0.01	− 0.09	0.08																					
Income	0.36	− 0.29	− 0.22	0.04	− 0.05	− 0.23	0.02	− 0.02																				
Married	0.20	− 0.31	− 0.02	0.05	− 0.05	0.00	0.02	0.00	0.45																			
Below HS	− 0.25	0.05	0.27	0.00	− 0.01	0.31	0.01	− 0.01	− 0.34	− 0.10																		
HighSch	− 0.08	0.10	0.06	− 0.04	− 0.01	0.04	0.00	− 0.01	− 0.32	− 0.15	− 0.10																	
SomColl	− 0.02	0.04	0.00	− 0.06	0.04	− 0.06	− 0.01	0.03	− 0.18	− 0.07	− 0.17	− 0.31																
Bache	0.11	− 0.06	− 0.09	0.04	− 0.01	− 0.07	− 0.01	0.00	0.26	0.11	− 0.14	− 0.26	− 0.42															
Graduate	0.12	− 0.10	− 0.10	0.07	− 0.02	− 0.06	0.02	− 0.01	0.41	0.15	− 0.11	− 0.21	− 0.34	− 0.29														
Town	0.07	− 0.02	− 0.10	− 0.03	0.07	− 0.11	0.01	0.08	− 0.06	− 0.01	− 0.04	0.03	0.05	− 0.02	− 0.04													
Sub urban	0.08	− 0.06	− 0.04	0.03	− 0.04	− 0.02	0.02	− 0.07	0.21	0.06	− 0.05	− 0.06	− 0.07	0.09	0.07	− 0.30												
Small urban	− 0.02	0.04	0.02	0.00	− 0.04	0.01	− 0.01	0.00	0.01	0.01	0.02	− 0.01	− 0.02	− 0.01	0.03	− 0.21	− 0.37											
Large urban	− 0.25	0.04	0.25	0.07	− 0.02	0.27	− 0.01	− 0.03	− 0.06	− 0.04	0.09	− 0.02	− 0.02	− 0.01	0.01	− 0.16	− 0.29	− 0.21										
Catholic	0.09	− 0.08	− 0.04	0.00	− 0.03	− 0.05	0.00	0.01	0.20	0.08	− 0.09	− 0.09	− 0.06	0.11	0.09	0.03	− 0.06	0.08	0.12									
Other Rel	0.08	− 0.04	− 0.04	− 0.04	− 0.02	− 0.04	0.00	0.04	0.10	0.06	− 0.06	− 0.06	− 0.03	0.04	0.07	0.03	− 0.05	0.08	0.01	− 0.10								
Other Priv	0.00	− 0.03	− 0.01	0.02	0.02	− 0.02	0.00	− 0.01	0.15	0.02	− 0.03	− 0.05	− 0.06	0.01	0.13	− 0.02	− 0.03	0.07	0.03	− 0.05	− 0.03							
Opp-in-home	0.06	0.01	− 0.07	− 0.04	0.03	− 0.13	0.02	− 0.01	0.04	0.03	− 0.09	− 0.05	0.00	0.02	0.07	0.03	− 0.03	0.01	− 0.05	− 0.02	− 0.04	0.02						
Opp-out-home	0.12	− 0.10	− 0.06	0.01	− 0.04	− 0.08	0.02	− 0.01	0.30	0.08	− 0.14	− 0.16	− 0.08	0.11	0.21	− 0.07	0.09	0.02	0.04	0.09	0.05	0.08	0.09					
Years teaching	0.18	− 0.09	− 0.14	0.01	− 0.04	− 0.13	0.01	0.06	0.11	0.05	− 0.09	− 0.04	0.01	0.02	0.06	0.08	− 0.03	0.00	− 0.07	0.02	0.06	0.01	0.00	0.04				
Trained teacher	0.05	0.01	− 0.05	− 0.02	− 0.01	− 0.08	− 0.01	0.06	0.01	− 0.01	− 0.04	− 0.01	0.01	0.03	0.00	0.03	− 0.01	0.01	− 0.06	− 0.01	− 0.06	− 0.01	0.01	0.01	0.00			
Science time	0.00	0.10	− 0.05	0.00	− 0.05	− 0.05	− 0.01	0.04	− 0.02	− 0.04	− 0.01	0.01	0.01	− 0.01	0.01	0.00	0.02	− 0.02	− 0.03	− 0.06	− 0.07	0.01	0.02	0.00	− 0.06	0.13		
Previous Math	0.27	− 0.27	− 0.15	0.07	− 0.06	− 0.12	0.11	0.05	0.43	0.22	− 0.23	− 0.19	− 0.09	0.16	0.24	− 0.05	0.10	0.02	− 0.02	0.05	0.07	0.08	0.02	0.18	0.09	0.02	0.00	
Previous Read	0.28	− 0.22	− 0.17	0.04	− 0.06	− 0.19	− 0.06	0.05	0.44	0.20	− 0.27	− 0.19	− 0.08	0.15	0.26	− 0.03	0.10	0.03	− 0.03	0.13	0.08	0.08	0.01	0.19	0.12	0.05	0.00	0.72

## Abbreviations

ECLS-K: Early Childhood Longitudinal Study, Kindergarten Class of 1998-99
SES: Socioeconomic status
STEM: Science, technology, engineering, and mathematics
